# IDH mutation and MGMT promoter methylation in glioblastoma: results of a prospective registry

**DOI:** 10.18632/oncotarget.5683

**Published:** 2015-10-12

**Authors:** Pei Yang, Wei Zhang, Yinyan Wang, Xiaoxia Peng, Baoshi Chen, Xiaoguang Qiu, Guilin Li, Shouwei Li, Chenxing Wu, Kun Yao, Wenbin Li, Wei Yan, Jie Li, Yongping You, Clark C. Chen, Tao Jiang

**Affiliations:** ^1^ Beijing Neurosurgical Institute, Capital Medical University, Beijing, China; ^2^ Department of Neurosurgery, Beijing Tiantan Hospital, Capital Medical University, Beijing, China; ^3^ Department of Epidemiology and Biostatistics, School of Public Health and Family Medicine, Capital Medical University, Beijing, China; ^4^ Department of Radiation Therapy, Beijing Tiantan Hospital, Capital Medical University, Beijing, China; ^5^ China National Clinical Research Center for Neurological Diseases, Beijing, China; ^6^ Department of Neuropathology, Beijing Neurosurgical Institute, Capital Medical University, Beijing, China; ^7^ Department of Neurosurgery, Beijing Sanbo Brain Hospital, Capital Medical University, Beijing, China; ^8^ Department of Pathology, Beijing Sanbo Brain Hospital, Capital Medical University, Beijing, China; ^9^ Department of Oncology, Beijing Shijitan Hospital, Capital Medical University, Beijing, China; ^10^ Department of Neurosurgery, the First Affiliated Hospital of Nanjing Medical University, Nanjing, China; ^11^ Center for Theoretic and Applied Neuro-Oncology, Division of Neurosurgery, University of California, San Diego, CA, USA

**Keywords:** glioblastomas, IDH, MGMT, temozolomide, radiation

## Abstract

**Background:**

The relative contribution of isocitrate dehydrogenase mutations (mIDH) and O6-methylguanine-DNA methyltransferase promoter methylation (methMGMT) as biomarkers in glioblastoma remain poorly understood.

**Methods:**

We investigated the association between methMGMT and *mIDH* with progression free survival and overall survival in a prospectively collected molecular registry of 274 glioblastoma patients.

**Results:**

For glioblastoma patients who underwent Temozolomide and Radiation Therapy, OS and PFS was most favorable for those with tumors harboring both *mIDH* and methMGMT (median OS: 35.8 mo, median PFS: 27.5 mo); patients afflicted glioblastomas with either *mIDH* or methMGMT exhibited intermediate OS and PFS (mOS: 36 and 17.1 mo; mPFS: 12.2 mo and 9.9 mo, respectively); poorest OS and PFS was observed in wild type IDH1 (wtIDH1) glioblastomas that were MGMT promoter unmethylated (mOS: 15 mo, mPFS: 9.7 mo). For patients with wtIDH glioblastomas, TMZ+RT was associated with improved OS and PFS relative to patients treated with RT (OS: 15.4 mo v 9.6 mo, *p* < 0.001; PFS: 9.9 mo v 6.5 mo, *p* < 0.001). While TMZ+RT and RT treated mIDH patients exhibited improved overall survival relative to those with wtIDH, there were no differences between the TMZ+RT or RT group. These results suggest that mIDH1 conferred resistance to TMZ. Supporting this hypothesis, exogenous expression of mIDH1 in independent astrocytoma/glioblastoma lines resulted in a 3–10 fold increase in TMZ resistance after long-term passage.

**Conclusion:**

Our study demonstrates IDH mutation and MGMT promoter methylation status independently associate with favorable outcome in TMZ+RT treated glioblastoma patients. However, these biomarkers differentially impact clinical TMZ response.

## INTRODUCTION

While randomized control trials (RCTs) form the cornerstone of modern evidence based medicine [1, 2], pre-defined trial inclusion and exclusion criteria often result in study populations that fail to represent the general patient population [3, 4]. As such, efforts to meaningfully impact clinical management require thoughtful integration of RCT results in the context of the prospective observational studies that provide data more representative of the general patient population [3, 4]. In this context, a major arm of the Chinese Glioma Genome Atlas (CGGA) was dedicated to a prospective registry that collected molecular profiles, radiographic response, and overall survival data for glioma patients treated in China.

Two molecular biomarkers of significant interest for glioblastoma involve isocitrate dehydrogenase (*IDH*) mutations and O6-methylguanine-DNA methyltransferase (MGMT) promoter methylation [5, 6]. IDHs are enzymes that catalyze the decarboxylation of isocitrate to α-ketoglutarate [7]. There are three isoforms of IDHs, termed IDH1, 2, and 3. Nearly all *IDH* mutations in glioblastomas involve substitution of R132 of *IDH1*, though rare mutations in R172 of *IDH2* are also reported [7, 8]. To simplify the terminology for the remainder of the manuscript, mutations in *IDH1* and *IDH2* will be referred to as mIDH. mIDH in glioblastoma simultaneously result in the loss of native enzymatic activity [9] as well as conferred novel activity in the production of 2 hydroxyglutarate (2HG) [10]. These enzymatic alterations ultimate trigger epigenetic changes [11] that defined the Glioma CpG Island Methylation phenotype (G-CIMP) [12], a phenotype that is associated with improved prognosis [8, 12, 13].

Another important biomarker in glioblastoma involves MGMT promoter methylation (methMGMT) [14, 15]. MGMT encodes an evolutionarily conserved DNA repair enzyme responsible for detoxifying temozolomide (TMZ) induced DNA damages [16, 17]. Clinically, high MGMT mRNA and protein expression has been associated with therapeutic resistance to DNA alkylating agents in a number of cancers [18, 19]. A major mechanism of MGMT regulation involves methylation of CpG islands in the promoter region [20]. Methylation of these regions suppresses MGMT transcription [21, 22]. mMGMT has been associated with favorable response to temozolomide in glioblastoma patients by two RCTs, including NOA-8 [23], and the Nordic Trial [24]. Interestingly, the EROTC-NCIC [25] demonstrated that MGMT promoter methylation additionally carried prognostic value in patients who did not receive TMZ.

While there is general consensus of the importance of mIDH and methMGMT as glioblastoma biomarkers, it remains unclear whether they present overlapping or independent clinical information as biomarkers. We analyzed our prospective CGGA registry to address this question.

## RESULTS

### Patient characteristics and treatment

Since the initiation of the prospective CGGA glioblastoma registry in 2004, a total of 151 subjects were enrolled by June of 2014 and molecular analysis performed. Given fulfillment of the needed sample size, we initiated analysis of this dataset. The patient characteristics were shown in Table 1. There were slightly more males (62%) than females in this group. The median age was 48 (range 18–81). 54% of the enrolled glioblastoma patient presented with preoperative KPS of ≥80. Gross total resection was achieved in 163 (59%) of the glioblastoma patients. Postoperative radiotherapy was performed in all patients enrolled in this study. TMZ chemotherapy was administered to 229 patients (84%), while 45 (16%) patients received postoperative radiotherapy without TMZ (RT). Of the 274 patients enrolled in this study, the status of IDH1/2 mutation and MGMT promoter methylation was analyzed in 229 glioblastomas. 31 glioblastomas were assessed only for IDH1/2 mutation, and 9 only for MGMT promoter methylation. mIDH1/2 was observed in 56 cases (21%). methMGMT was observed in 95 cases (40%). The IDH1 and MGMT promoter methylation status of the study populations were: 32 (14%) mIDH and methMGMT. 54 (25%) wild type IDH (wtIDH1) and methMGMT, 15 (7%) mIDH and unmethMGMT patients, and 128 (56%) wtIDH and unmethMGMT patients.

**Table 1:** Clinical and molecular characteristics

Variable Total (n, %)		GBM 274
**Age, years**	Median (range)	48 (18–81)
	Age ≥45	166 (61)
	Age <45	108 (39)
**Gender**	Male	169 (62)
	Female	105 (38)
**Preoperative KPS score**	Preoperative KPS ≥80	149 (54)
	Preoperative KPS <80	125 (46)
**Resection**	Gross total resection	163 (59)
	Subtotal	111 (41)
**Postoperative Treatment**	RT plus TMZ	229 (84)
	RT only	45 (16)
**IDH1/2 mutation**	Mutation	56 (20)
	Wildtype	209 (76)
	NA	9 (3)
**MGMT promoter methylation**	Methylated	95 (35)
	Not methylated	143 (52)
	NA	36 (13)

### Progression free and overall survival

**Figure 1:**
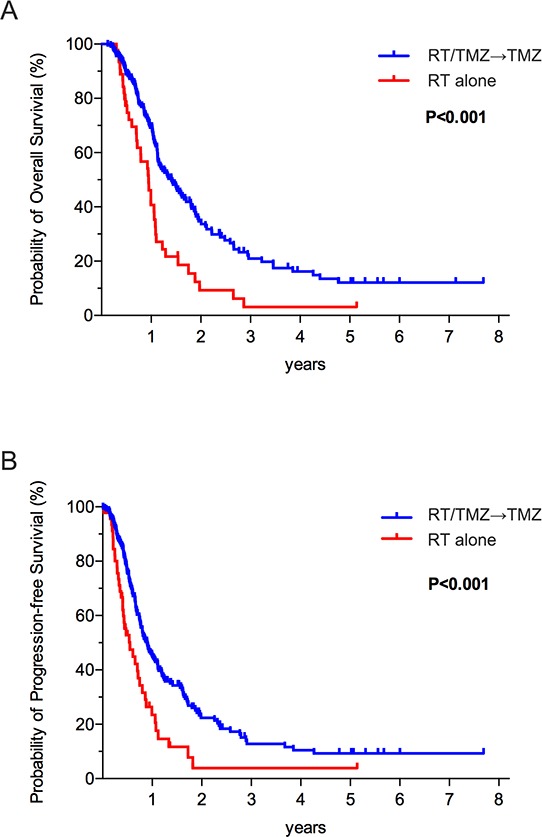
**The Kaplan–Meier estimates for overall survival A.** and progression-free survival **B.** indicated that patients underwent RT plus TMZ treatment exhibited much longer survivals than did who received RT only.

**Figure 2:**
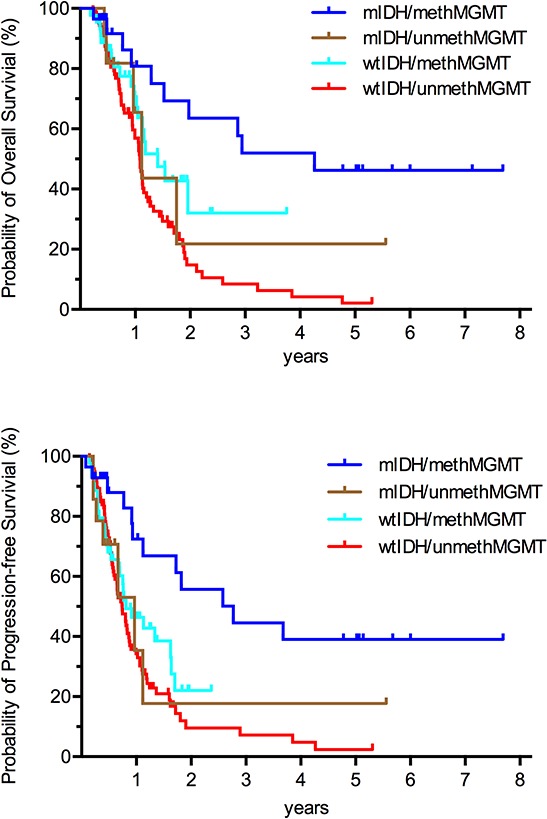
Kaplan–Meier curves showing that, among GBMs, patients with both IDH mutation and MGMT promoter methylation exhibited the best prognosis; patients harboring either mutated IDH or methylated MGMT promoter exhibited intermediate prognosis; patients with wild-type IDH and unmethylated MGMT promoter exhibited the worst

For the study population, the 6-month, 1-, 3-, and 5-year OS were 85, 65, 17 and 10%, respectively. The 6-month, 1-, 3-, and 5-year PFS were 67, 39, 11 and 8%, respectively (Supplemental Table 1). Both OS and PFS were more favorable in patients who underwent TMZ treatment (Figure 1A and 1B). Kaplan–Meier estimates of OS and PFS for the GBM patients according to IDH mutation and MGMT promoter methylation were respectively shown in Supplemental Figure 1. Consistent with previous publications [8, 26, 27], patients with mIDH glioblastomas exhibited longer survival relative to those with wild type IDH (Supplemental Figure 1A, *p* = 0.024 and Supplemental Figure 1B, *p* = 0.03). And for those underwent different postoperative treatment, patients with mIDH also exhibited longer survival relative to patients with wild type IDH in RT plus TMZ group and RT alone group, respectively (Supplemental Figure 2A and 2B). Favorable survival patterns were also observed in patients with methMGMT glioblasotmas relative to those with unmethylated MGMT Promoter (Supplemental Figure 1C, *p* = 0.005 and Supplemental Figure 1D, *p* = 0.028). When both IDH mutation and MGMT promoter methylation status were considered, the patients exhibiting the longest OS and PFS were those with mIDH1 and methMGMT (35.8 and 27.5 mos, respectively). Patients harboring either mIDH1 or methMGMT exhibited intermediate OS and PFS (14.1 and 9.4 mos, respectively). Patients with wtIDH1 and unmethMGMT exhibited the worst PFS and OS (13.2 and 8.9 mos, respectively). The differences between these groups were statistically significant (Figure 2A and 2B). In a multivariate model that accounted for the age at diagnosis and pre-operative KPS, both mIDH mutation (HR 0.46, 95% CI 0.26–0.82, *p* = 0.008) and methMGMT (HR 0.63, 95% CI 0.42–0.95, *p* = 0.028) independently associated with improved OS (Table 2).

**Table 2:** Variables related to OS in GBMs undergoing TMZ/RT: Univariate and multivariate Cox analyses

Variable (*n* = 229)	OS				
	Univariate Cox analysis	Multivariate Cox analysis			
	*P* value	Hazard ratio	95% CI		*P* value
			Lower	Upper	
**Age**	0.001	1.019	1.002	1.036	0.03
**Preoperative KPS**	<0.001	0.969	0.956	0.983	<0.001
**IDH1 mutation**	<0.001	0.444	0.227	0.869	**0.018**
**MGMT methylation**	<0.001	0.594	0.371	0.949	**0.03**

### Effect of TMZ in mIDH glioblastoma patients

**Figure 3:**
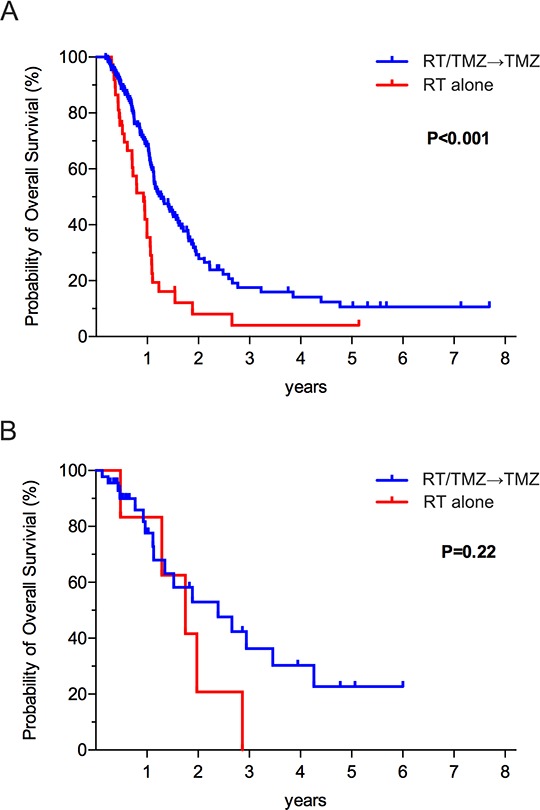
**A.** The Kaplan–Meier estimates for overall survival indicated that the group of GBM patients with wt IDH who were randomly assigned to the RT plus TMZ treatment groups exhibited significantly longer survival than did the group who were randomly assigned to RT only. **B.** Among GBM patients with IDH mutation, a more favorable survival benefit was not observed in the RT plus TMZ treatment group compared to the RT alone group.

The clinical association between methMGMT and TMZ sensitivity is well documented [15, 28]. In contrast, whether IDH status bears relevance to clinical TMZ sensitivity remains an open question. Because the patients in our registry had the choice of undergoing TMZ+RT or RT alone, we were able to examine how TMZ impacted the survival pattern of patients with wt and mIDH1 glioblastomas. For patients with wtIDH, Patients who underwent RT+TMZ exhibited significantly longer survival times compared to the patients who were assigned to the RT alone treatment (Figure 3A, *p* < 0.001), supporting the efficacy of TMZ in this patient population; the median survivals were 15.4 months for the RT+TMZ treatment group and 11.2 months for the RT alone group. The 2-year survival rates were 28% and 8%, respectively. However, among mIDH1 patients, the survival pattern of patients undergoing RT+TMZ or RT were comparable (median survivals: 29.1 vs. 21.3 months; 2-year survival rates: 47% vs. 21%, respectively; Figure 3B, *p* = 0.22). Similar results were observed after controlling for MGMT promoter methylation status (Supplemental Table 2). Among the GBM patients with either methMGMT or unmethMGMT, a more favorable survival benefit was observed in the RT+TMZ treatment group compared to the RT alone group (Supplemental Figure 3). In aggregate, these outcome data suggest that IDH mutation status may influence glioblastoma sensitivity to TMZ.

### Expression of mIDH1 induced relative resistance to TMZ

**Figure 4:**
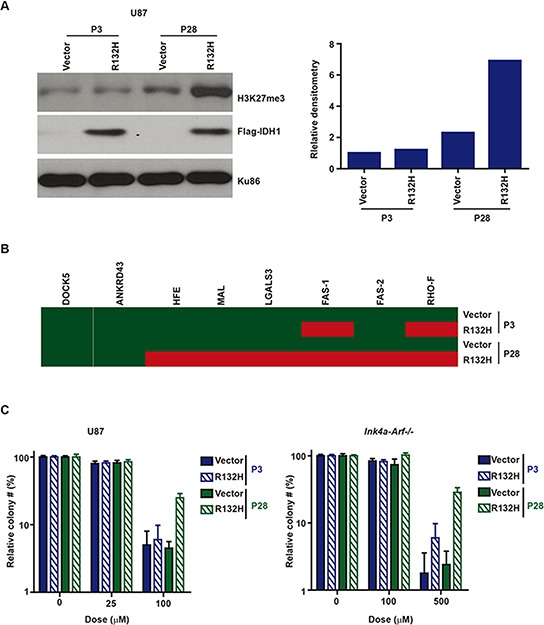
A. Prolonged passage after IDH1-R132H expression increased chromatin deposition of H3K27me3 in human U87MG glioblastoma and murine *Ink4a/Arf−/−* astrocytic cells Cell lysates from short (5 passages) and long-term passaged (28 passages) cells were prepared, fractionated by gel-electrophoresis, and probed with an anti-H3K27me3 (Abcam#6002), anti-Flag (Sigma#8592), or anti-Ku86 (Santa Cruz#sc-1485) antibody. Right: Densitometry quantitation of the immuno-blot. The H3K27me3 signal was normalized to the Ku86 signal. **B.** Prolonged passage after IDH1-R132H expression in human U87 glioblastoma cells induced altered DNA methylation patterns from a G-CIMP- pattern to a G-CIMP+ pattern. Red indicates that the genomic region of interest is methylated. Green indicates the lack of DNA methylation. **C.** Prolonged passage after IDH1-R132H expression increased TMZ resistance of human U87MG glioblastoma and murine *Ink4a/Arf−/−* astrocytic cells. Clonogenic survival was assessed 14 days after TMZ treatment. Please note that the Y-axis is plotted on a log-scale.

We wished to test whether exogenous expression of mIDH1 decreased cellular sensitivity to TMZ. Consistent with previously published results, short-term (5 passages) expression of IDH1-R132H [29] in the human U87MG glioblastoma line or the murine *Ink4a-Arf−/−* astrocytic line did not significantly alter the epigenetic landscape [11]. However, prolonged passage (28 passage) of the cell with IDH1-R132H expression resulted in global epigenetic changes, reflected by increased chromatin H3K27me3 deposition (Figure 4A) [11] and the DNA methylation patterns consistent with the G-CIMP phenotype (Figure 4B) [12]. In early passaged cells, U87MG or *Ink4a-Arf−/−* cells expressing mIDH1-R132H exhibited TMZ sensitivity comparable to those expressing the vector. However, after 28 passages, mIDH1-R132H expressing cells exhibited a 3–10 fold increase in TMZ resistance relative to vector expressing cells or early passaged IDH1-R132H expressing cells (Figure 4C). These results suggest that prolonged expression of IDH1-R132H confer cellular resistance to TMZ.

## DISCUSSION

The importance of IDH mutations and MGMT promoter methylation as biomarkers for glioblastoma patient is widely recognized [5, 6, 8, 15, 30]. However, the relative contribution of each biomarker to clinical outcome remains poorly understood. Whether the effects of one biomarker masked that of the other or the two biomarkers independently associated with clinical outcome remains an open question. The results of our prospectively collected molecular registry support the latter hypothesis. Our results show that glioblastoma patients harboring various combinations of mIDH and methMGMT exhibit significant differences in OS and PFS. Patients afflicted with glioblastomas harboring both mIDH and methMGMT exhibited the longest OS and PFS while those with neither showed the shortest OS and PFS. Patients with glioblastomas harboring either mIDH or methMGMT showed intermediate survival. These results are largely consistent with the observation that the biologic function of mIDH and methMGMT appear distinct [5, 6]. The former modulate global epigenetic alterations [11], and the latter influence the expression of the DNA repair protein, MGMT [15].

Mutant IDH1 has been proved *in vitro* that may drive a unique set of transformative events that indirectly enhance HR and facilitate repair of temozolomide-induced DNA damage and temozolomide resistance [31]. However, it is important to note that there is currently little clinical information in terms of how IDH mutation status decreased clinical response to TMZ. The landmark Stupp study [25] that conclusively demonstrated the clinical efficacy of TMZ was conducted without stratification for IDH status, as this study was completed before the discovery of IDH mutations [32]. Keeping in mind a large body of work suggesting that mIDH glioblastomas are fundamentally different (on a molecular and clinical level) relative glioblastomas with wild type IDH, caution in extrapolating the Stupp results to mIDH glioblastoma is warranted. Here, we took advantage of an unique opportunity to address this question. Between 2005 and 2014, glioblastoma patients in China were strongly encouraged to undergo TMZ + RT; but, patients were ultimately given the opportunity to refuse TMZ. Many patient refused TMZ because of insurance coverage. In this context, we were able to analyze cohort of TMZ+RT and RT treated patients and show that the efficacy of TMZ is notably better against wild type IDH glioblastomas relative to the IDH mutated tumors. Consistent with this observation, we demonstrated that mutation of IDH lead to increased TMZ resistance. On the basis of our study on temozolomide chemotherapy in GBMs, we observed significant relationship between outcome and IDH mutations. wtIDH tumor group benefit much more than mIDH tumor group from temozolomide chemotherapy. This may suggest that in biological behavior and etiopathology, IDH1 wild-typed tumors are more malignant and invasive that has been proved getting a statistically significant and clinically meaningful survival benefit with the addition of temozolomide to radiotherapy early in the course. [33] In the contrary, glioblastomas with IDH1 mutation might be kin to lower grade gliomas with benigner biological behaviors and less chemotherapy sensitivity.

The relative insensitivity of mIDH1 glioblastoma carry significant implication in terms of the glioblastoma treatment paradigm, particularly in the context of the ever-expanding number of potential tumoricidal and immuno-therapies specifically targeting IDH mutations [34–36]. Moreover, TMZ treatment has been shown to facilitate mutagenesis and promote the emergence of recurrence or more aggressive clone [37–39]. Therapeutic use of TMZ in patients harboring glioblastomas with intrinsic resistance may be problematic in this context. Finally, it is desirable to avoid the toxicity profiles of TMZ in patients who will unlikely benefit from treatment [40]. In these contexts and given the results observed in our registry, there is a critical need to re-evaluate the use of TMZ in patients afflicted with IDH mutated gliomas. Careful evaluation of this issue is warranted since the inherent favorable prognosis of mIDH patients may be mis-interpreted as the therapeutic effect of TMZ.

Given the prospective design of the study, our results are subject to all the biases inherent of a non-randomized, prospective study. There are two particular limitations that warrant further discussion. First, as a non-randomized study, the predictive value of mIDH may be explained by selection bias in terms of patients undergoing RT versus TMZ+RT. We believe that this bias is unlikely since TMZ+RT was universally recommended to all glioblastoma patients in China since 2005. Because of initial unfamiliarity of the Chinese neurosurgeon to TMZ, patients were given the opportunity to decline therapy. Until TMZ was covered by insurance, many patients declined therapy for financial reasons while others declined because they did not wish to be the first to receive a new therapy in China. Given that the choice of therapy was made by the patient, the influence of selection bias on the part of the physician should be minimized. Notably, the age and KPS of patients undergoing RT only and TMZ+RT in our study were comparable. Second, validation of our observation in an independent dataset is warranted prior to generalizations that impact clinical care or trial enrollment recommendations.

## MATERIALS AND MEHTODS

### Patients enrollment and assessment of clinical outcome

As a part of the Chinese Glioma Genome Atlas (CGGA) project (http://www.cgga.org.cn/portal.php), we prospectively consented patients who underwent surgical resection for malignant gliomas at the Glioma Treatment Center of Beijing Tiantan Hospital and Beijing Sanbo Brain Hospital from October 2004 to February 2014. The study was approved by the ethics committee in both hospitals and written informed consent was obtained from each patient. All of data and samples were collected under the IRB of Beijing Tiantan Hospital and Beijing Sanbo Brain Hospital. The criteria of enrollment include: age more than 18 years-old, and histologically confirmed glioblastoma and patient's consent. The histological diagnosis was assessed by two independent neuropathologists and graded according to the 2007 World Health Organization (WHO) classification. [41] Clinical data, including the patient's age at diagnosis, sex, presenting symptoms, preoperative Karnofsky Performance Status (KPS) score, operation status, and MR imaging were collected. Specimens were collected after definitive diagnosis and stored as paraffin embedded blocks and snap-frozen for subsequent molecular characterization. The collected specimen were verified by our pathologists to harbor >80% viable tumor tissue. For each enrolled patient, clinical follow-up was performed every 6 months and surveillance MRI performed every 3 months.

### Treatment

Maximal tumor resection while preserving the key eloquent cortex was the principle goal during surgery. The extent of resection was assessed on the postoperative enhanced MRI within 72 h and graded as gross total or subtotal resection. Patients subsequently underwent concomitant TMZ and RT (TMZ+RT) or radiotherapy in addition to concomitant and adjuvant TMZ chemotherapy (RT+TMZ) or postoperative radiotherapy only (RT only). Postoperative adjuvant radiotherapy was routinely delivered to the patient within four weeks after surgery. The total dose was 54–60 Gy, which was divided into 30 daily fractions of 1.8–2 Gy each, and five fractions were administered per week. For the patients who received adjuvant chemotherapy, the treatment was administered four weeks after radiation, and at least two cycles of chemotherapy were administered. Concomitant chemotherapy consisted of oral TMZ at a daily dose of 75 mg/m [2] that was given seven days per week from the first to the last day of radiotherapy for at most 49 days. After a four-week break, the patients received adjuvant oral TMZ (150–200 mg/m2) for five days every 28 days. A total of six cycles of TMZ chemotherapy were administered if no disease progression or irreversible hematological toxic effects were observed.

### DNA extraction and molecular evaluation

Genomic DNA was isolated from the frozen tumor tissues using the QIAamp DNA Mini Kit (Qiagen) according to the manufacturer's protocol. The DNA concentration and quality were measured using the Nano-Drop ND-1000 spectrophotometer (NanoDrop Technologies, Houston, TX). For the subpopulations for which biomaterials were available, the IDH1/2 mutation (DNA pyro-sequencing) and MGMT promoter methylation (DNA pyro-sequencing) status were assessed based on previously published methods [42].

### Surveillance and follow-up

Survival data were collected in the clinics during the patient visits. The patients who underwent only tumor biopsy were not followed up at our center and were therefore excluded from the survival analysis. The baseline examinations included CT and magnetic resonance imaging (MRI), full blood counts and blood chemistry tests and physical examinations. During radiotherapy (with or without TMZ), the patients were seen every week. Twenty-one to 28 days after the completion of radiotherapy and every three months thereafter, the patients underwent comprehensive evaluations that included physical examinations and radiologic assessments of the tumors. During the adjuvant TMZ therapy, the patients underwent monthly clinical evaluations and comprehensive evaluations at the end of cycles 3 and 6. Overall survival (OS) was defined as the time between surgery and death. Progression-free survival (PFS) was defined based on the RANO criteria [43].

### Construction of isogenic IDH1-R132H expressing astrocytic and glioblastoma lines

Human glioma cells U87MG are purchased from American Type Culture Collection (Manassas, VA). Murine *Ink4a-Arf−/−* cells were kindly provided by Dr. Oren Becher (Duke University Medical Center). The cells were propagated at 37°C (humidified atmosphere containing 5% CO2) in Dulbecco's modified Eagle medium supplemented with 10% fetal calf serum, 2 mM L-glutamine, 100 U/mL penicillin G sodium, and 100 mg/mL streptomycin sulfate (Gibco) [44, 45]. The wild-type human IDH1 and IDH1-R132H mutant (c.395G > A) were generously provided by Dr. Kun-Liang Guan (University of California, San Diego) and Yue Xiong (Fudan University, China). The constructs were confirmed by Sanger sequencing. Retrovirus packaging and infection were performed as previously described and stably infected cells were generated by selection with G418 (600 μg/ml) for 2 weeks [29]. Stably infected cells were passage by splitting every 3–4 days and stored in liquid nitrogen. Cells isolated after passage 5 and passage 28 were further analyzed for TMZ sensitivity [46] and epigenetic changes. G-CIMP DNA methylation studies were performed as described by Noushmehr et al. [12]. Eight markers were tested for G-CIMP DNA methylation. Each marker was coded as red if methylated and green if unmethylated. One of these markers (DOCK5) is unmethylated in CIMP, whereas the remaining seven markers show G-CIMP-specific hypermethylation. G-CIMP-positive status was determined if ≥6 of the 8 genes had G-CIMP-defining hyper- or hypomethylation. H3K27me3 (Abcam) immunostaining was performed as previously described [29].

### Statistical analyses

The hypothesis that patients with mIDH and/or methMGMT exhibit longer median survival time than patients without these genetic alternations is based on the published literature [8, 14, 15, 26, 27, 47]. The aggregate of these publications suggest that mIDH is present in approximately 15% of the general glioblastoma population (based on general prevalence of primary and secondary glioblastomas in the general population as well as the prevalence of mIDH in primary and secondary glioblastomas). The hazard ratio of survival (calculated based on published median survival) for wtIDH patient relative to mIDH patient was 0.51 [8, 26]. methMGMT is expected to be present in approximate 45% of the general glioblastoma population. The hazard ratio of survival for unmethMGMT patients relative to that of methMGMT patients (calculated based on published median survival) was approximately 0.65 [15, 28]. Using these parameters, we estimate that a minimum of 240 glioblastoma patients will be needed (α of 0.05 and β of 0.8) to determine the relative contribution of mIDH and methMGMT as prognostic factors. Sample size calculations were performed under the PASS software (version 11.0). The comparison of median survival time between different treatment, different subtype of clinicopathological factors was mapped with the Kaplan–Meier analysis and compared with the two-sided of log-rank test. Then, a Cox proportional-hazards model was performed to examine the predictive values of the clinicopathological factors after adjusting the confounding of age and preoperative KPS score. Statistical significance level was defined as *P* < 0.05.

## CONCLUSIONS

Our study demonstrates that IDH and MGMT promoter methylation status independently associate with favorable outcome in TMZ+RT treated glioblastoma patients. However, these biomarkers differentially impact the likelihood of clinical TMZ response. Validation of these results can fundamentally alter treatment paradigms for patients afflicted with IDH mutated glioblastomas.

## SUPPLEMENTARY FIGURES AND TABLES


