# Supplementary material for: IDH mutation and MGMT promoter methylation in glioblastoma: results of a prospective registry
Source: Oncotarget. 2015 Oct 12;6(38):40896–906. doi: 10.18632/oncotarget.5683 (PMC4747376; doi:10.18632/oncotarget.5683)
Supplement: Supplementary file 1 [file oncotarget-06-40896-s001.pdf]

## SUPPLEMENTARY FIGURES AND TABLES

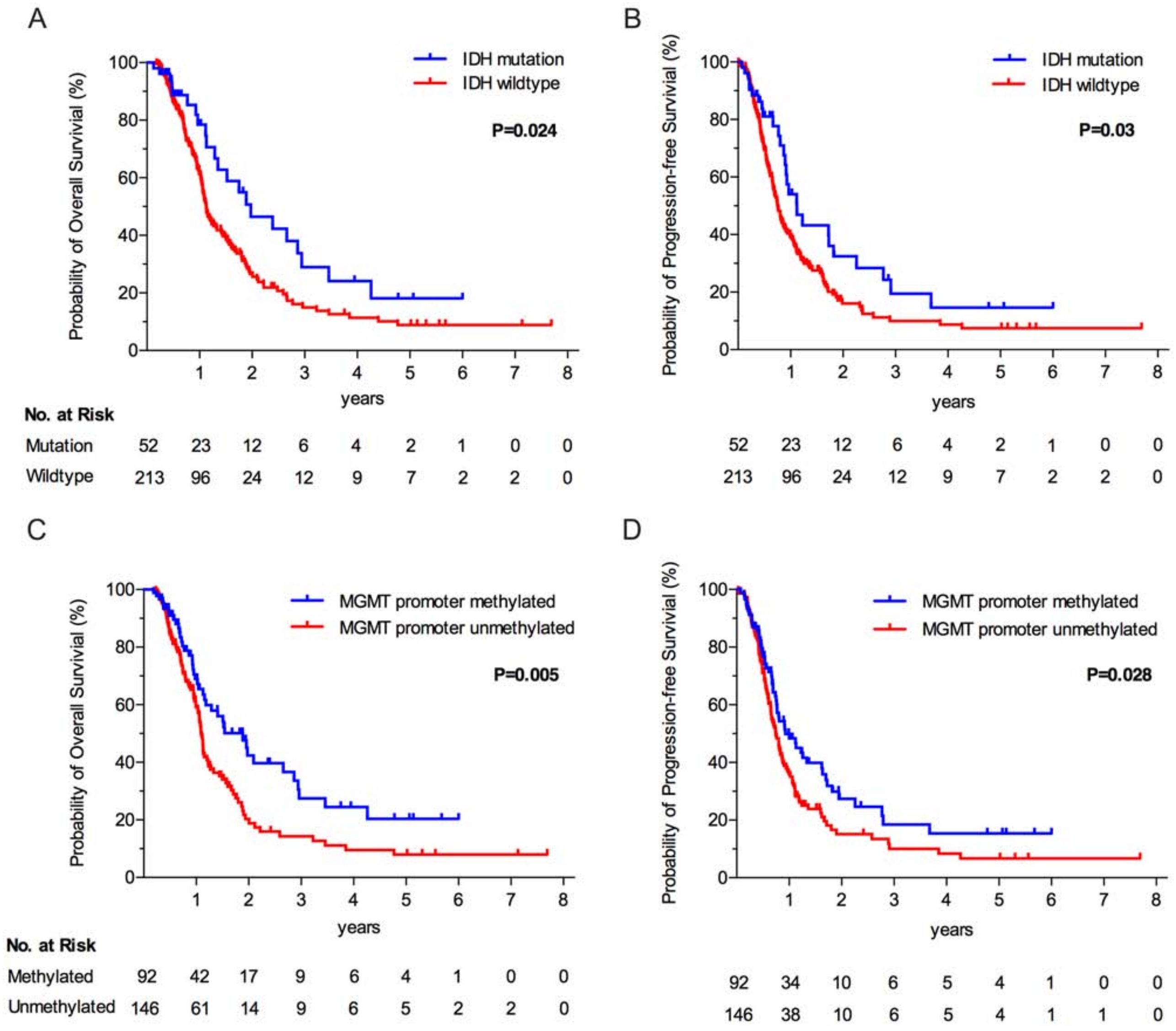


**Supplementary Figure S1: Kaplan–Meier curves showing that IDH mutation was prognostic of longer overall survival A.** and progression-free survival **B.** among the GBM patients. Meanwhile, MGMT promoter methylation was also a prognostic factor for both longer overall survival **C.** and progression-free survival **D.** among GBM patients.

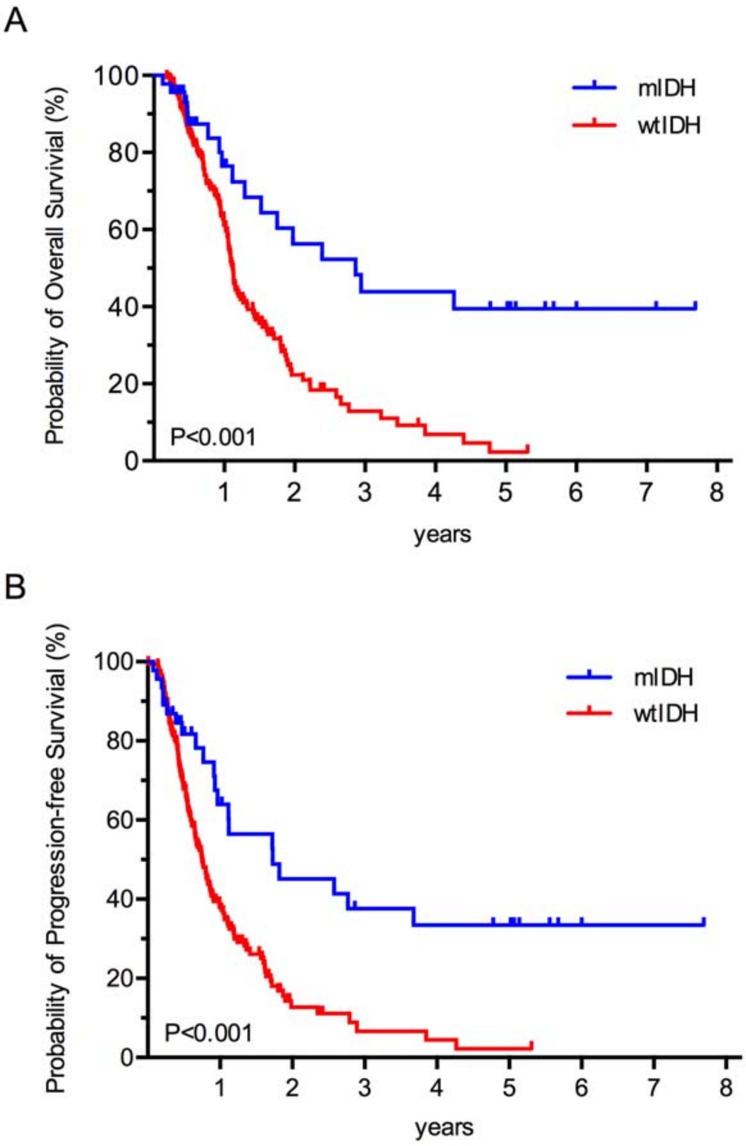


**Supplementary Figure S2: A.** The Kaplan–Meier estimates for overall survival indicated that the group of GBM patients with IDH1 mutation who underwent RT plus TMZ treatment exhibited a longer survival time than the patients with IDH mutation that received RT only treatment. **B.** The GBM patients with IDH wild type who underwent RT plus TMZ treatment also exhibited a better prognosis than those with IDH1 wild type receiving RT only.

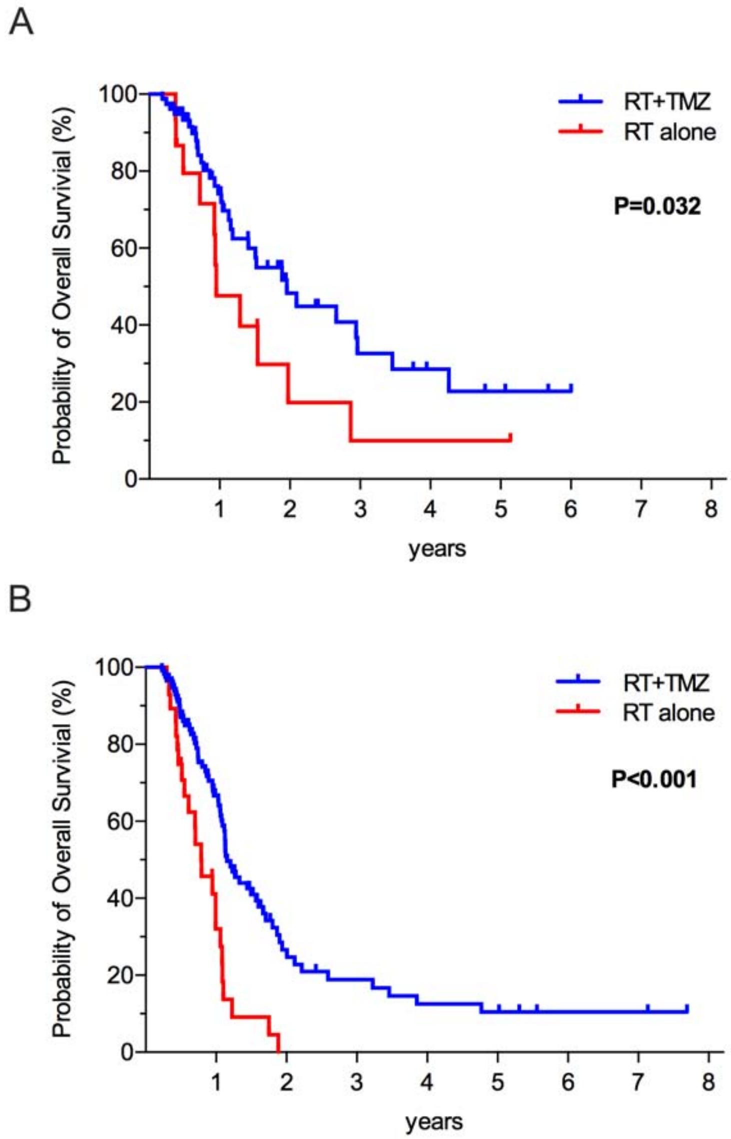


**Supplementary Figure S3: A.** The Kaplan–Meier estimates for overall survival indicated that the group of GBM patients with MGMT promoter methylation who underwent RT plus TMZ treatment exhibited a longer survival time than the patients with MGMT promoter methylation that received RT only treatment. **B.** The GBM patients with unmethylated MGMT promoters who underwent RT plus TMZ treatment also exhibited a better prognosis than those with unmethylated MGMT promoters receiving RT only.

## Supplementary Table S1: Survival According to Treatment

| Treatment group | Glioblastoma | | |
|---|---|---|---|
| | Total | RT/TMZ → TMZ | RT |
| No. of patients | 274 | 229 | 45 |
| Median OS (month) | 14.5 | 17.8 | 11.4 |
| at 6-month (%) | 85 | 89 | 70 |
| at 1-year (%) | 65 | 69 | 34 |
| at 3-year (%) | 17 | 22 | 3 |
| at 5-year (%) | 10 | 12 | 3 |
| Median PFS (month) | 9.8 | 10.7 | 6.5 |
| at 6-month (%) | 67 | 71 | 47 |
| at 1-year (%) | 39 | 43 | 20 |
| at 3-year (%) | 11 | 13 | 4 |
| at 5-year (%) | 8 | 9 | 4 |

## Supplementary Table S2: Survival According to IDH, MGMT and Treatment

| MGMT | IDH | Treatment group | No. of patients | Median OS (days) | *P* value | Median PFS (days) | *P* value |
|---|---|---|---|---|---|---|---|
| **MGMT promoter methylation** | **IDH mutation** | **RT+TMZ** | 26 | 1074 | 0.082 | 824 | 0.065 |
| | | **RT alone** | 6 | 720 | | 409 | |
| | **IDH wildtype** | **RT+TMZ** | 46 | 514 | 0.04 | 296 | 0.061 |
| | | **RT alone** | 8 | 338 | | 157 | |
| **MGMT promoter unmethylation** | **IDH mutation** | **RT+TMZ** | 14 | 1080 | NA | 365 | NA |
| | | **RT alone** | 1 | NA | | NA | |
| | **IDH wildtype** | **RT+TMZ** | 101 | 449 | <0.001 | 292 | 0.001 |
| | | **RT alone** | 27 | 287 | | 239 | |
